# The Relation of Imperfect Teeth to Cataract

**Published:** 1875-05

**Authors:** 


### ARTICLE XI.

### *The Relation of Imperfect Teeth to Cataract.*

In an article on this subject, Mr. Jonathan Hutchinson, F. R. C. S., gives his views as follows:

By the way of summary, I think it may be stated—

1. That it is exceptional to meet with lamellar cataracts, excepting in association with an imperfect development of the enamel of the teeth ; but that definite exceptions, in which the teeth are quite perfect, do occur.

2. That the kind of defect observed in the teeth consists in the absence of the enamel, and is shown in the incisors, canines, and first molars of the *permanent* set, to the almost invariable exemption of the præmolars. That, for purposes of diagnosis, the first molars are by far the most important, and may rank as the test teeth, since they not unfrequently show the defect when the others escape.

3. That it is highly probable that the defects in the development of the teeth are usually due to the influence of mercury exhibited in infancy; although it is quite possible that other influences, attended perhaps by inflammation of the gums, may occasionally produce similar results.

4. That teeth of the kind alluded to are met with very often in persons who are not the subjects of zonular cataract.

5. That the very important observation made by Arlt, that the subjects of lamellar cataract have usually suffered from convulsions in infancy, is fully borne out by further examination; and that it is very unusual to find lamellar cataract without such history.

6. That it is probable that there is a direct connection between the occurrence of convulsions in infancy and the development of lamellar cataract.

7. That, whilst there is every reason to believe that the defective teeth which are met with in connection with lamellar cataract are the results of mercury, the evidence seems opposed to the belief that the lenticular opacity is also due to the influence of the drug. The great frequency of mercurial teeth without lamellar cataract, and the not very infrequent occurrence of lamellar cataract, without mercurial teeth, are opposed to this view.

8. That the very frequent ceincident occurrence of lamellar cataract with defective teeth, is to be explained by reference to the frequency with which mercury is given for the treatment of convulsions in infancy.

9. That there is no reason whatever for supposing that lamellar cataracts have any connection with hereditary syphilis.

10. That, whilst it is certainly true that lamellar-cataracts are commonly met with in young persons who show general defects of development, short statue, ill-shaped heads, defective intellect, dwarfed lower jaws, or other physiognomical peculiarities, yet there is seldom any proof of the existence of rickets; whilst it is quite possible that the peculiarities mentioned may be due to the disturbance of the nervous system in infancy in connection with the convulsions.

11. It is very important to distinguish between mercurial teeth and syphilitic teeth, and the peculiarities presented by each usually render this easy; the two are, however, as might have been expected, not uncommonly met with together.—*British Medical Journal.*

